# Supplementary material for: Inhibition of Phytopathogenic and Beneficial Fungi Applying Silver Nanoparticles In Vitro
Source: Molecules. 2022 Nov 23;27(23):8147. doi: 10.3390/molecules27238147 (PMC9738576; doi:10.3390/molecules27238147)
Supplement: Supplementary file 1 [file molecules-27-08147-s001.zip › molecules-1967384-supplementary.pdf]

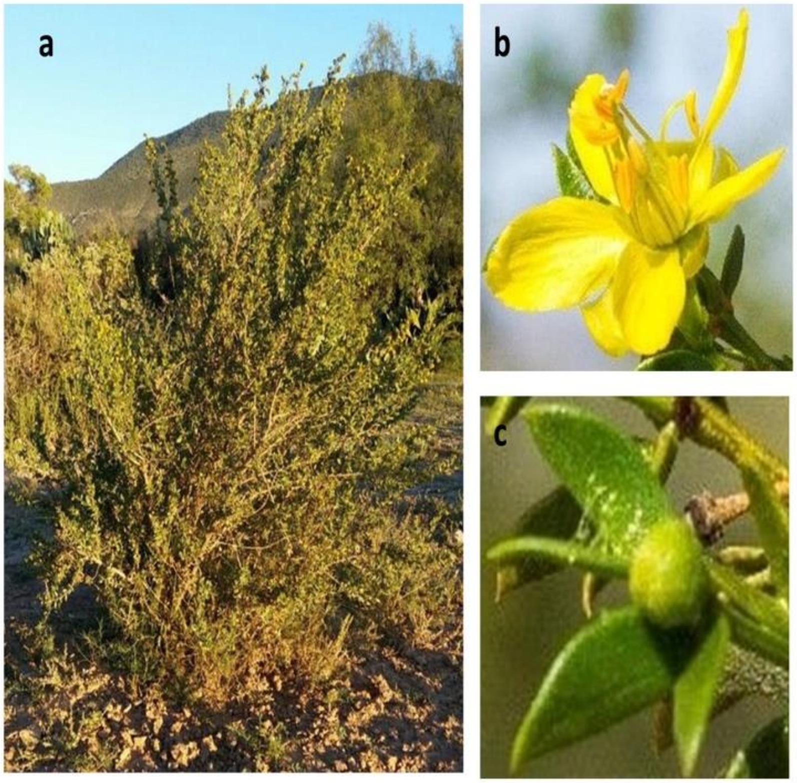


Figure S1. a) *Larrea tridentata* plant, b) *L. tridentata* flower, c) *L. tridentata* leaf and fruit.

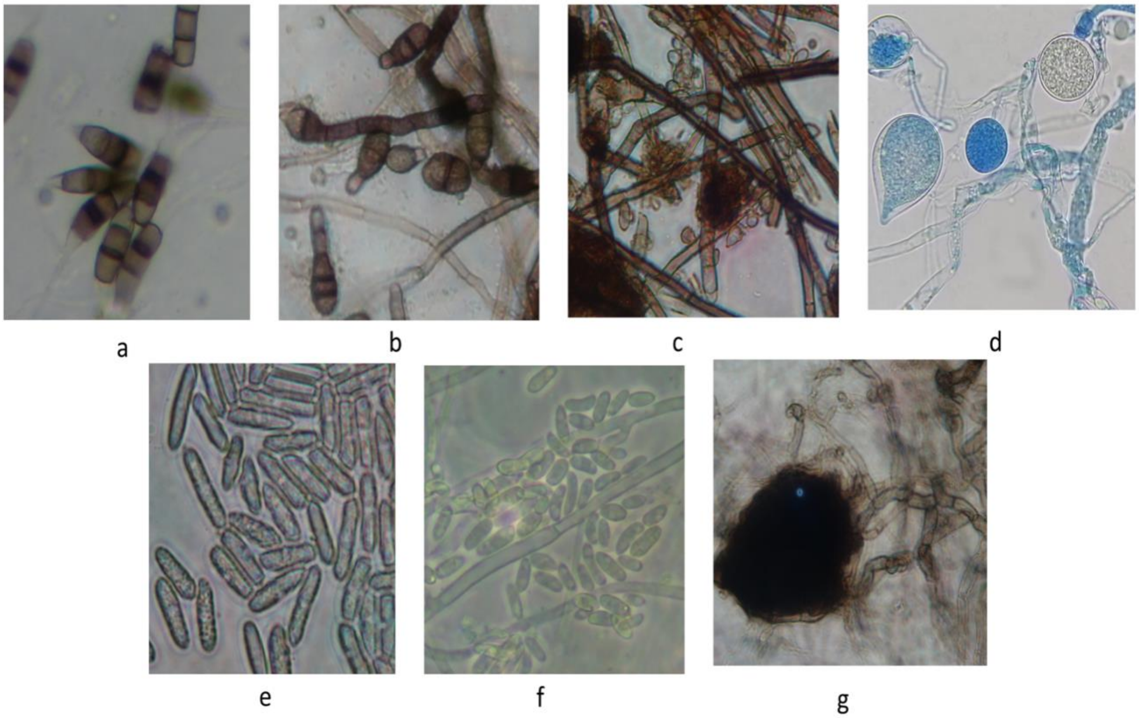


Figure S2. a) Conidia of *Pestalotia* sp., b) Conidia of *Alternaria solani*, c) Conidiophores and conidia of *Botrytis cinerea*, d) Sporangium of *Phytophthora* sp., e) Conidia of *Colletothrichum gloesporoides*, f) Microconidia of *Fusarium oxisporum*, and g) Mycelium and sclerotium of *Macrophomina* sp. This was examined at 40X magnification under a compound binocular microscope (Carl ZEISS Axio Scope A1).
